# Tirzepatide-induced ketoacidosis with hyperglycemia in a patient without diabetes

**DOI:** 10.20945/2359-4292-2026-0028

**Published:** 2026-04-01

**Authors:** Mendel Shloush, Vania Rodriguez, Victor Guillen, Diego Lugo Baruqui

**Affiliations:** 1 Florida International University Herbert Wertheim College of Medicine, Miami, Florida, USA; 2 Department of Internal Medicine, Mount Sinai Medical Center, Miami Beach, Florida, USA

**Keywords:** Tirzepatide, ketoacidosis, GLP-1 agonist, GIP, obesity pharmacotherapy, weight loss, adverse drug reaction

## Abstract

Tirzepatide,a dual GLP-1 and GIP receptor agonist, is increasingly used for weight management in both patients with diabetes and patients without diabetes. While gastrointestinal side effects such as nausea are common, severe metabolic complications like ketoacidosis are rare and often overlooked. We report the case of a 38-year-old woman with congenital heart disease who developed acute ketoacidosis with hyperglycemia following five months of tirzepatide therapy. Laboratory findings confirmed high anion gap metabolic acidosis, elevated ketones, and significant hyperglycemia. With supportive care, including intravenous fluids, insulin infusion, and electrolyte replacement, she recovered fully. This represents a rare case of tirzepatide-induced hyperglycemic ketoacidosis in a patient without diabetes. In contrast, the two previously reported cases by Singh and cols. and Iqbal and cols. involved patients without diabetes who developed euglycemic ketoacidosis with normal glucose levels while on tirzepatide. This case underscores the importance of vigilant monitoring for metabolic complications, including hyperglycemia and ketoacidosis, in patients without diabetes, particularly those with comorbidities.

## INTRODUCTION

With obesity rates rising globally, new medications like tirzepatide are transforming weight management. Tirzepatide is increasingly utilized in both patients with diabetes and patients without diabetes for weight control (^1^,^2^). This once-weekly injectable targets GLP-1 and GIP receptors to reduce appetite, delay gastric emptying, and enhance glucose-dependent insulin release. It is effective for weight loss and glycemic control (^3^), though most adverse effects are gastrointestinal, including nausea, vomiting, and diarrhea (^4^).

Rare metabolic complications such as euglycemic ketoacidosis (EKA), characterized by acidosis and ketone accumulation despite normal blood glucose, have been reported. Two recent cases described EKA in women without diabetes using tirzepatide (^5^,^6^). We present a distinct case of a patient without diabetes with congenital heart disease who developed *hyperglycemic* diabetic ketoacidosis (DKA) while on tirzepatide. To our knowledge, this represents the first reported case of hyperglycemic DKA in a patient without diabetes associated with tirzepatide use.

## CASE PRESENTATION

A 38-year-old woman with a history of bicuspid aortic valve and repaired aortic coarctation arrived at the emergency department with two days of nausea, vomiting, abdominal pain, and constipation. She had no fever, diarrhea, or unusual dietary changes and had eaten very little in the prior 48 hours. She had been on Zepbound (tirzepatide) for five months, gradually increasing to 7.5 mg weekly, for weight management. Over the five-month span on tirzepatide, her weight dropped from 86 kg to 72 kg. She did not have a history of diabetes, glucose metabolism disorders, alcohol abuse, or eating disorders, and had never experienced acidosis before. She reported a family history of Hashimoto’s thyroiditis in a maternal aunt and a first cousin. Her only allergy was to amoxicillin. On arrival, her vital signs were: temperature 97.4°F, heart rate 135 bpm, BP 177/102, respiratory rate 17 breaths per minute, spO_2_ of 100 on room air, and BMI 26.7. She was dehydrated but alert and oriented. Her abdomen was moderately tender in the left lower quadrant, with no signs of peritonitis. A CT scan of the abdomen and pelvis showed no obstruction, perforation, or other acute pathology (**Table 1)**.

**Table 1:** Initial laboratory findings on admission

Parameter	Value	Reference range
WBC	27,000/µL	4,000-11,000
Hemoglobin	16.9 g/dL	12.0-15.5 g/dL
Hematocrit	51.6%	35-45%
Platelets	381,000/µL	150,000-400,000/µL
Neutrophils	92%	40-75%
Chloride	116 mmol/L	98-106 mmol/L
Phosphorus	2.0 mg/dL	2.5-4.5 mg/dL
Potassium	5.4 mmol/L	3.5-5.0 mmol/L
Bicarbonate (CO_2_)	10.6 mmol/L	22-29 mmol/L
Glucose	285 mg/dL	70-110 mg/dL
Anion gap	26.4 mmol/L	8-16 mmol/L
Lactate	4.7 mmol/L	0.5-2.2 mmol/L
Beta-hydroxybutyrate	62 mmol/L	<0.6 mmol/L
Total protein	9.9 g/dL	6.0-8.3 g/dL
HbA1c	5.1%	≤ 5.6%
Urine Glucose	> 1000 mg/dL	Negative
Creatinine	1.2 mg/dL	0.55-1.02 mg/dL
BUN	9 mg/dL	7-18 mg/dL
AST/ALT	0.66	0.7-1.2
ABG (PCO_2_)	< 18 mmHg	35-45 mmHg
ABG (pH)	7.13	7.35-7.45
Urine Ketones	> 150	Negative

Given her high anion-gap metabolic acidosis, elevated ketones, and hyperglycemia, we considered various differentials, including lactic acidosis, ketoacidosis (diabetic, alcoholic, or starvation), and toxin ingestion (methanol, ethylene glycol, salicylates), as well as renal failure. Lactic acidosis was present but insufficient alone to explain the degree of acidosis (lactate 4.7 mmol/L); renal function was near normal; toxic alcohol and salicylate levels were not clinically suspected based on history and presentation; and there was no evidence of sepsis or shock. Alcoholic ketoacidosis was excluded by her negative alcohol history, and starvation ketosis alone seemed unlikely to account for the severity of metabolic derangement, though her recent poor intake may have contributed or precipitated the event in conjunction with tirzepatide’s appetite-suppressing effects. After these considerations, our leading diagnosis was tirzepatide-associated ketoacidosis with hyperglycemia. She also had a Naranjo Adverse Drug Reaction Probability scale of 3, indicating a possible ADR.

She was admitted to the Intensive Care Unit and management followed the current standard diabetic ketoacidosis (DKA) protocol, including aggressive intravenous fluid resuscitation to correct dehydration, continuous insulin infusion to reduce blood glucose levels and suppress ketogenesis, and careful electrolyte monitoring, particularly potassium replacement to prevent hypokalemia. Frequent laboratory assessments guided adjustments in therapy until metabolic parameters normalized, including blood glucose levels consistently below 200 mg/dL, closure of the anion gap (less than 12 mEq/L), serum bicarbonate levels rising above 18 mEq/L, and absence of ketones in serum. These criteria guided the safe transition from intravenous insulin to subcutaneous therapy and ultimately marked clinical and biochemical recovery. She was discharged on day 5 with instructions to stop tirzepatide and follow up with endocrinology.

After discharge, the patient underwent additional outpatient evaluation to assess for an underlying predisposition to ketosis and to exclude LADA-type diabetes. IA-2, glutamic acid decarboxylase 65, and ZnT8 antibodies were all within normal limits, and a repeat hemoglobin A1c was 5.3%, indicating preserved long-term glycemic control. While no Oral Glucose Tolerance Test was performed, and serum insulin and C-peptide were not measured, the completed tests argue against autoimmune-mediated beta-cell dysfunction or chronic insulin deficiency, making an underlying insulinopenic state unlikely in this patient.

## DISCUSSION

Euglycemic ketoacidosis involves high anion gap acidosis and elevated ketones without significant hyperglycemia (^7^). While often linked to SGLT2 inhibitors (^7^-^11^), it has now been seen with GLP-1 and dual GLP-1/GIP agonists like tirzepatide (^5^,^6^). In this case, the patient had two days of severe nausea, vomiting, and abdominal pain, during which she had little food intake. This reduced food intake, likely due to tirzepatide’s appetite suppression as well as known side effects, triggered fat breakdown and ketone production, similar to starvation states (^12^). Unlike diabetic ketoacidosis, which results from severe insulin deficiency, this ketoacidosis can occur with relative insulin sufficiency, allowing partial ketone suppression (^8^). This case differs from those by Singh and cols. (^5^) and Iqbal and cols. (^6^), who reported EKA with normal glucose levels (5.6 mmol/L and 4.9 mmol/L, respectively). Singh and cols. described a 48-year-old woman with EKA after titrating to 5 mg weekly (^5^). Iqbal and cols. reported a 21-year-old with no comorbidities who presented with EKA (^6^). Our patient, however, had a glucose of 285 mg/dL, despite a normal HbA1c of 5.1% and no diabetes history.

To our knowledge, this is the first reported case of hyperglycemic ketoacidosis in a patient without diabetes following tirzepatide use. The explanation for hyperglycemia in this patient may include increased stress hormones like cortisol. In fact, Weiss and cols. reported rare cases of extreme stress hyperglycemia in the setting of acute illness (^13^). Dungan and cols. also described hyperglycemia due to stress-induced counter-regulatory hormones, such as cortisol and epinephrine, which promote hepatic glucose production and insulin resistance in acute illness (^14^). This interplay of stress-induced hyperglycemia and tirzepatide-driven ketogenesis gives a possible explanation to the novel presentation of hyperglycemic ketoacidosis in this patient without diabetes.

In addition to reduced oral intake and stress-related hyperglycemia, another theoretical explanation is the presence of polymorphisms in the GIP receptor, which could lead to excessive stimulation of glucagon secretion and relative suppression of insulin secretion. In this scenario, the observed hyperglycemia might be driven primarily by increased hepatic glucose production rather than by peripheral insulin resistance or caloric restriction alone. Although this mechanism is speculative and was not directly assessed in this patient, including it provides a biologically plausible pathophysiologic explanation and strengthens the overall clinical interpretation.

However, the definitive cause of hyperglycemia in our patient is uncertain. This case expands the known risks of tirzepatide, showing ketoacidosis can occur later in treatment, in complex patients, and with varying glucose levels. Her high white blood cell count and lactate initially suggested sepsis, but no fever, infection, or abnormal imaging supported this. Antibiotics were discontinued once a non-infectious cause was clear.

The patient was successfully treated for tirzepatide-induced hyperglycemia using the standard diabetic ketoacidosis (DKA) protocol, which included intravenous fluids, insulin infusion, and electrolyte management, leading to normalization of glucose levels, anion gap closure, and resolution of ketosis. However, this case underscores the emerging challenge of managing DKA presentations linked to GLP-1 receptor agonists like tirzepatide, which may manifest with atypical features such as euglycemia or variable glycemic patterns.

These observations highlight the potential need for developing updated or adjunctive treatment protocols tailored specifically for GLP-1-associated DKA to optimize patient outcomes and guide clinicians in managing these increasingly recognized presentations. Potential changes include earlier and more frequent monitoring of ketones regardless of glucose levels, more cautious insulin dosing to avoid hypoglycemia, and a greater emphasis on identifying and correcting precipitating factors unique to GLP-1 therapies, such as dehydration from gastrointestinal side effects. Additionally, protocols might incorporate extended monitoring after initial resolution due to the prolonged pharmacodynamic effects of these agents. These adaptations aim to improve detection, treatment safety, and outcomes in this evolving clinical context.

While the patient’s poor oral intake undoubtedly contributed to her ketosis, the direct pharmacological effects of tirzepatide on metabolic pathways, independent of starvation, must be considered, especially given the patient’s hyperglycemia. The drug’s dual agonism of GLP-1 and GIP receptors alters insulin and glucagon secretion, potentially disrupting the balance that prevents excessive ketogenesis. Given the limited number of reported cases on this topic, the exact mechanism by which tirzepatide directly induces ketoacidosis is not yet fully understood. Further research is warranted to understand the direct pharmacological effects of this class of drugs on ketone body metabolism, especially in patients without diabetes.

## CONCLUSION

Tirzepatide is highly effective for weight loss and glycemic control, but its appetite-suppressing effects can lead to serious metabolic complications. Clinicians should consider ketoacidosis in patients on GLP-1 or GIP therapies presenting with nausea, vomiting, or acidosis, regardless of diabetes status or glucose levels. This case highlights the importance of vigilant monitoring, especially in patients with complex medical histories, to distinguish from euglycemic cases and ensure timely treatment.

## Data Availability

the datasets used and/or analyzed during the current study available from the corresponding author on reasonable request.

## References

[1] Nauck MA, Quast DR, Wefers J, Pfeiffer AFH. (2021). The evolving story of incretins (GIP and GLP-1) in metabolic and
cardiovascular disease: A pathophysiological update. Diabetes Obes Metab.

[2] Sinha R, Papamargaritis D, Sargeant JA, Davies MJ. (2023). Efficacy and Safety of Tirzepatide in Type 2 Diabetes and Obesity
Management. J Obes Metab Syndr.

[3] Jastreboff AM, Aronne LJ, Ahmad NN, Wharton S, Connery L, Alves B, SURMOUNT-1 Investigators (2022). Tirzepatide Once Weekly for the Treatment of
Obesity. N Engl J Med.

[4] Trujillo J. (2020). Safety and tolerability of once-weekly GLP-1 receptor agonists in
type 2 diabetes. J Clin Pharm Ther.

[5] Singh R. (2025). Euglycemic Ketoacidosis in a Non-diabetic Patient After
Tirzepatide Use: A Cautionary Tale. Cureus.

[6] Iqbal PMR, Maadarani OS, Bitar ZI. (2024). Tirzepatide-Induced Ketoacidosis in Non-Diabetic
Patients. Eur J Case Rep Intern Med.

[7] Rosenstock J, Ferrannini E. (2015). Euglycemic Diabetic Ketoacidosis: A Predictable, Detectable, and
Preventable Safety Concern With SGLT2 Inhibitors. Diabetes Care.

[8] Diaz-Ramos A, Eilbert W, Marquez D. (2019). Euglycemic diabetic ketoacidosis associated with sodium-glucose
cotransporter-2 inhibitor use: a case report and review of the
literature. Int J Emerg Med.

[9] Plewa MC, Bryant M, King-Thiele R. (2023). Euglycemic Diabetic Ketoacidosis.

[10] Mistry S, Eschler DC. (2020). Euglycemic Diabetic Ketoacidosis Caused by SGLT2 Inhibitors and a
Ketogenic Diet: A Case Series and Review of Literature. AACE Clin Case Rep.

[11] Peters AL, Buschur EO, Buse JB, Cohan P, Diner JC, Hirsch IB. (2015). Euglycemic Diabetic Ketoacidosis: A Potential Complication of
Treatment With Sodium-Glucose Cotransporter 2 Inhibition. Diabetes Care.

[12] Cahill GF Jr (2006). Fuel metabolism in starvation. Annu Rev Nutr.

[13] Weiss SL, Alexander J, Agus MS. (2010). Extreme stress hyperglycemia during acute illness in a pediatric
emergency department. Pediatr Emerg Care.

[14] Dungan KM, Braithwaite SS, Preiser JC. (2009). Stress hyperglycaemia. Lancet.

